# The role of inner nuclear membrane protein emerin in myogenesis

**DOI:** 10.1096/fj.202500323

**Published:** 2025-04-03

**Authors:** Nicholas Marano, James M. Holaska

**Affiliations:** ^1^ Department of Biomedical Sciences Cooper Medical School of Rowan University Camden New Jersey USA; ^2^ Rowan‐Virtua School of Translational Biomedical Engineering and Sciences Stratford New Jersey USA

**Keywords:** emerin, Emery‐Dreifuss muscular dystrophy, laminopathy, myogenic differentiation

## Abstract

Emerin, a ubiquitously expressed inner nuclear membrane protein, plays a central role in maintaining nuclear structure and genomic organization, and in regulating gene expression and cellular signaling pathways. These functions are critical for proper myogenic differentiation and are closely linked to the pathology of Emery‐Dreifuss muscular dystrophy 1 (EDMD1), a laminopathy caused by mutations in the *EMD* gene. Emerin, along with other nuclear lamina proteins, modulates chromatin organization, cell signaling, gene expression, and cellular mechanotransduction, processes essential for muscle development and homeostasis. Loss of emerin function disrupts chromatin localization, causes dysregulated gene expression, and alters nucleoskeletal organization, resulting in impaired myogenic differentiation. Recent findings suggest that emerin tethers repressive chromatin at the nuclear envelope, a process essential for robust myogenesis. This review provides an in‐depth discussion of emerin's multifaceted roles in nuclear organization, gene regulation, and cellular signaling, highlighting its importance in myogenic differentiation and disease progression.

## Graphical abstract

Emerin function is critical for proper genome reorganization and temporal gene expression necessary for terminal myogenic differentiation. Emerin interacts with both HDAC3 and histone methyltransferases (HMTs) to organize chromatin at the inner nuclear membrane. Upon differentiation induction, the genome reorganizes to mediate the activation or repression of commitment or cell cycle genes, respectively. This is coordinated by the interaction of emerin with HDAC3 and HMTs at the nuclear envelope.

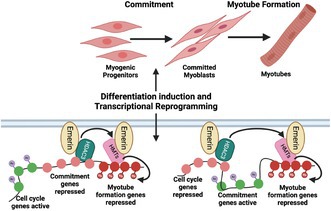

## 1 INTRODUCTION

Emerin is a ubiquitously expressed inner nuclear membrane (INM) protein with reported functions in nuclear structure,^1^, ^2^, ^3^ mechanotransduction,^4^, ^5^ cell signaling,^6^, ^7^, ^8^, ^9^ genomic organization, and gene expression.^10^, ^11^, ^12^, ^13^ Emerin function is critical in regulating nuclear structure and genomic organization in skeletal muscle and cardiac muscle.^14^ Mutations in emerin cause tissue‐specific disease, indicating a vital role for emerin in facilitating skeletal muscle and cardiac muscle function and homeostasis. Here, we discuss the role of emerin in myogenic differentiation.

The X‐linked form of EDMD (EDMD1) is caused by mutations in *EMD*.^15^ The pathology of EDMD was first described in a family of affected males exhibiting progressive skeletal muscle weakness, joint contractures, and myocardial abnormalities.^16^ EDMD results in cardiac arrhythmias, which can be fatal.^17^ The prevalence of EDMD1 is estimated to be between 1:500 000^18^ and 1:100 000 male births.^19^ Other forms of EDMD have been identified and are caused by mutations in *LMNA* (EDMD2/3),^20^ *SYNE‐1*/*SYNE‐2* (EDMD4/5),^21^ *FHL1* (EDMD6),^22^ and *TMEM43* (EDMD7).^23^

Most EDMD1 patients lack any detectable emerin expression. However, a small number of EDMD1 patients harbor unique emerin mutations which result in normal emerin expression levels and proper INM localization.^15^, ^24^, ^25^, ^26^, ^27^ These ‘special’ emerin mutants are point mutations or short deletion mutations that disrupt binding to various emerin‐binding partners. These include emerin mutants S54F,^15^ Q133H,^26^ Δ95‐99,^24^ and P183H.^25^ These mutants show disrupted binding to transcription regulators and nucleoskeletal proteins, depending on the mutation.^28^ Importantly, these ‘special mutants’ have been shown to disrupt myogenic differentiation similarly to emerin‐null cells.^27^ Notably, patients harboring these ‘special’ emerin mutations still exhibit the dystrophic phenotype.^15^, ^24^, ^25^, ^26^ Thus, studying the dysfunction of these specific emerin mutants will be important for elucidating the mechanisms driving EDMD1 disease pathology. More recently, three EDMD1‐causing emerin mutations in the LEM domain^29^, ^30^, ^31^ and two novel frameshift mutations in emerin have been observed.^32^

There are multiple hypotheses regarding the underlying cause of EDMD1: the nuclear structure hypothesis and genome regulation hypothesis, which are not mutually exclusive. Skeletal muscle and cardiac muscle undergo high burdens of mechanical stress, and mutations or reduction of INM proteins like emerin likely compromise nuclear integrity to increase the likelihood of nuclear rupture upon high shear stress. Supporting the genomic regulation hypothesis, emerin mutations disrupt genomic organization^33^ and gene expression,^11^, ^27^ which lead to impaired myogenic differentiation. Collectively, growing evidence suggests that both structural deficits and genomic organization deficits at the INM likely contribute to disease pathology.

## 2 EMERIN

Emerin is a 254‐residue, serine‐rich, type II integral inner nuclear membrane (INM) protein that is expressed in most mammalian tissues.^34^ The *EMD* gene (formerly known as *STA*) encodes emerin and is located in the distal region of Xq28.^15^ Emerin contains an evolutionarily conserved 40‐residue LEM (LAP2, emerin, MAN1) domain at its N‐terminus.^35^, ^36^ LAP2, emerin, and MAN1 are the founding members of the LEM‐domain family of proteins. Emerin bears sequence homology to the INM proteins LAP2β and MAN1,^37^ particularly in their LEM‐domain. The LEM domain binds to barrier‐to‐autointegration factor (BAF) which nonspecifically binds DNA via a helix‐hairpin‐helix motif.^38^, ^39^ Emerin and other LEM‐domain proteins are proposed to indirectly tether chromatin at the nuclear envelope (NE) through the interaction with BAF.^40^ LEM‐domain proteins may also share other functions with emerin, including regulation of nuclear structure and transcription.^41^

Emerin contains an 11‐residue luminal domain at the C‐terminus, preceded by a hydrophobic 23‐residue transmembrane domain.^15^, ^42^ Early studies showed the transmembrane domain of emerin was targeted to the endoplasmic reticulum (ER)^43^, ^44^ and that regions within the nucleoplasmic domain of emerin were responsible for its nuclear retention.^43^ Emerin is post‐translationally inserted into the ER via the TRC40/GET‐pathway (guided entry of tail anchored protein).^45^ Once integrated into the ER, emerin diffuses throughout the membrane of the ER, which is contiguous with the outer nuclear membrane (ONM). Similarly to other INM proteins, emerin likely diffuses through either peripheral or both central and peripheral channels in the nuclear pore complex (NPC).^46^ Emerin then arrives at its final destination at the INM and is retained, at least in part, by directly binding lamin A^47^ according to the diffusion‐retention model.^48^ Both the lamin A‐binding domain and transmembrane domain of emerin are required for emerin localization to the INM.^49^ Emerin mutants that fail to bind lamin A also fail to localize to the NE.^44^, ^49^ Mouse embryonic fibroblasts (MEFs) lacking A‐type lamins (*LMNA* ^−/−^) showed reduced retention of emerin in the NE and mislocalization of emerin to the ER,^50^, ^51^ which was rescued by the expression of lamin A in *LMNA* ^*−/−*^ fibroblasts.^52^ Partial retention of emerin at the NE in the absence of lamin A^13^ is likely due to interactions with other INM proteins such as lamin B receptor (LBR)^53^ or components of the linker of nucleoskeleton and cytoskeleton (LINC) complex.^4^, ^54^, ^55^

The nucleoplasmic region of emerin contains a large intrinsically disordered region (IDR).^56^, ^57^ The protein‐folding prediction software AlphaFold 3 supports emerin as an intrinsically disordered protein (IDP).^58^ NMR analysis also supports emerin as a highly disordered protein.^56^, ^59^ IDPs harbor amino acid sequences that lack a stable tertiary structure.^60^ IDPs or proteins with IDRs are capable of adapting different conformations to dynamically associate with various partners.^60^, ^61^ Emerin was shown to self‐assemble via its nucleoplasmic region, and some EDMD‐causing emerin mutations within the IDR disrupt this self‐assembly.^56^ The IDR of emerin facilitates binding to most of its protein partners,^28^ including nucleoskeletal components,^47^, ^62^ transcriptional regulators,^63^, ^64^, ^65^ and histone deacetylase 3 (HDAC3).^10^ We predict that emerin's IDR allows it to serve as a signaling node, allowing it to dynamically interact with itself or other protein partners. It will be important to determine whether binding to these partners causes emerin to adopt distinct conformations. Emerin is also highly post‐translationally modified,^66^, ^67^, ^68^, ^69^ so the effects of post‐translational modifications on emerin IDR structure and the interaction of IDR with specific partners also need to be defined.

## 3 MYOGENIC DIFFERENTIATION

**FIGURE 1:**
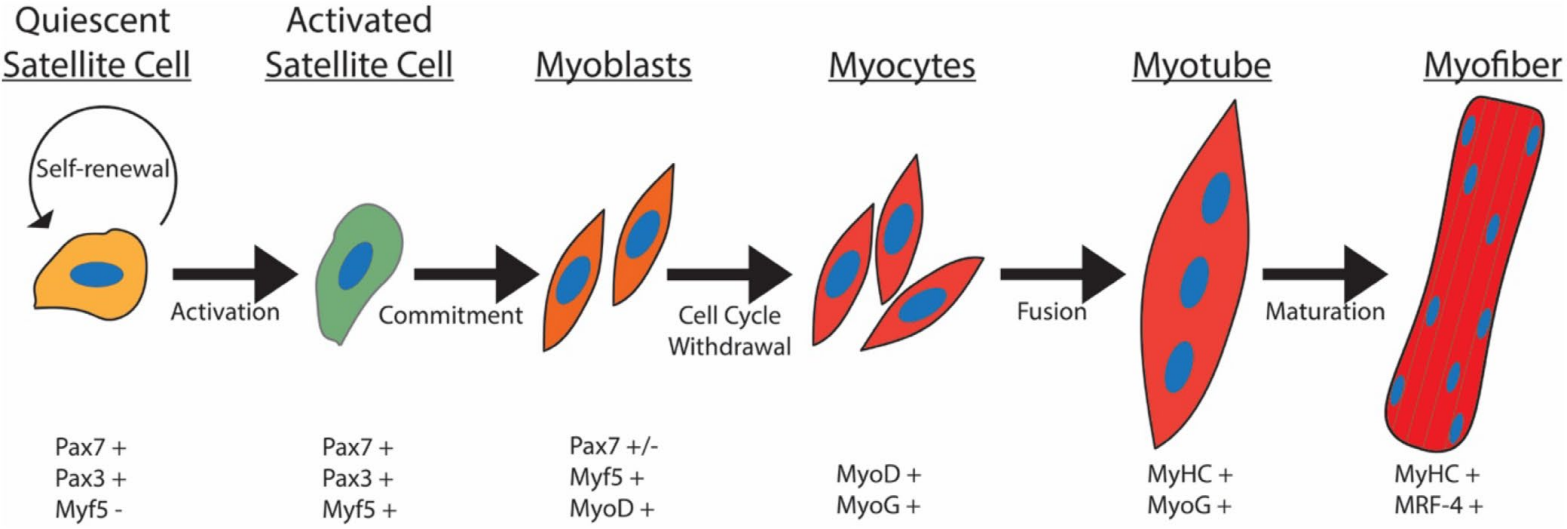
The myogenic differentiation program. Upon injury, quiescent satellites divide asymmetrically with one cell (Myf5^−^) replenishing the stem cell niche, while the other cell (Myf5^+^) becomes an activated satellite cell. Activated satellite cells continue differentiating to become committed myoblasts and begin expressing MyoD, while downregulating Pax7 expression. Myoblasts then exit the cell cycle to form myocytes, which begin expressing myogenin. Myocytes will then align and fuse into myotubes. Myotubes then mature and become integrated into myofibers.

In skeletal muscle regeneration, quiescent stem cells are activated to proliferate asymmetrically,^70^ with one cell maintaining the stem cell niche and the other cell committing to the myogenic lineage. Skeletal muscle stem cells (also called myogenic progenitors) are called satellite cells due to their location on the skeletal muscle fiber. Satellite cells are defined by the expression of paired box protein 3 (Pax3) and Pax7 (Figure 1) and the absence of transcription factor myogenic regulatory factor 5 (Myf5).^70^ Pax3 and Pax7 regulate cellular identity, where Pax7 is required for the commitment of myogenic precursors.^71^ Pax3 drives myogenic identity, as it functions as an activator of *Myf5* transcription and expression of Myf5 protein.^72^, ^73^ Activated stem cells will asymmetrically divide so that one cell replenishes the satellite cell niche (Pax7^+^/Myf5^−^), while the other becomes a committed myogenic progenitor (Figure 1), now defined as Pax7^+^/Myf5^+^.^70^ These committed myogenic progenitors then proliferate, differentiate, and fuse to themselves and the injured myofiber^74^, ^75^ to regenerate skeletal muscle.

Factors termed myogenic regulatory factors (MRFs) play a crucial role in differentiation.^76^, ^77^ MRFs are responsible for the activation of downstream myogenic lineage genes, which drive the differentiation program (Figure 1). These committed myogenic progenitor cells are defined by the expression of the transcription factor Myf5 and are primed for differentiation.^78^, ^79^ Myf5 activates the downstream expression of many early myogenic differentiation genes, including the myoblast determination protein 1 (MyoD).^80^ Activated satellite cells then become committed myoblasts by the expression of the MyoD transcription factor.^76^ MyoD is responsible for driving the expression of the myogenic differentiation program and is sufficient to convert fibroblasts to the myogenic lineage.^81^ Myogenin (MyoG) expression is activated by MyoD^82^ and regulates the myoblast to myocyte transition and myotube fusion.^83^ The temporal regulation and expression of MRFs are the major drivers of myogenic differentiation.^84^

As the myogenic differentiation program proceeds, the genome is dynamically reorganized,^33^, ^76^, ^85^ presumably to modulate the coordinated temporal expression of differentiation genes. Combinatorial lamin B1 immunofluorescence and 3D fluorescent in‐situ hybridization (3D‐immunoFISH) of MRF loci in differentiating myogenic progenitors were employed to analyze genomic reorganization.^33^ 3D FISH is a technique used to visualize the spatial localization of genomic loci in three dimensions.^86^ Myogenic progenitors lacking emerin were unable to temporally reorganize *Pax3*, *Pax7*, *Myf5*, and *MyoD1* at the NE during differentiation, resulting in the failure to temporally activate or repress *Pax3*, *Pax7*, *Myf5*, or *MyoD1* transcription.^33^ Further, ChIP (chromatin immunoprecipitation)‐qPCR using emerin antibodies in proliferating wild‐type myogenic progenitors showed enrichment of *MyoD1* and *Myf5* loci with emerin, while the *MyoD1* and *Myf5* loci lost peripheral localization in nuclei of emerin‐null cells.^33^ Additionally, RNA‐seq of differentiating wild type, emerin‐null, and EDMD1 mutant myogenic progenitor lines exhibits expression profiles consistent with ChIP‐qPCR and 3D‐immunoFISH datasets.^11^ This data supports a critical role for emerin in the organization and maintenance of myogenic differentiation genes at the NE.

## 4 GENOMIC ORGANIZATION

**FIGURE 2:**
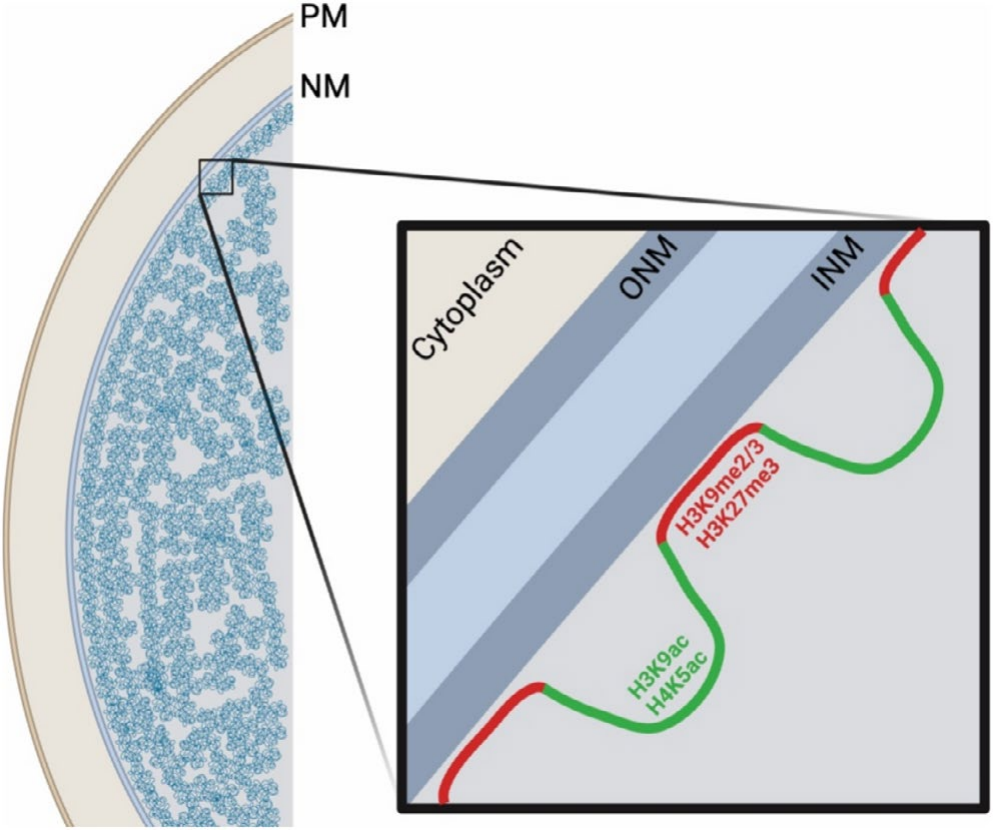
Lamina‐associated domains (LADs). Chromatin organized at the nuclear lamina are termed LADs. LADs at the nuclear envelope are enriched for the repressive histone modifications H3K9me2/3 and H3K27me3 (red). Non‐LAD regions are shown (green) and possess transcriptionally active epigenetic marks, notably H3K9ac and H4K5ac. Figure created with BioRender.com.

Genomic organization within the nucleus is vital for cellular identity, as it drives the expression of genes that are responsible for distinct cellular functions. The spatial organization of chromatin controls the activation or repression of specific genes.^87^, ^88^ Hi‐C techniques have been employed to investigate bulk chromatin interactions in topologically associated domains (TADs) and to generate 3D genomic maps.^89^ Hi‐C mapping revealed that chromatin is organized into regions with active or repressed transcription.^90^ These chromatin regions within the nucleus are divided into two compartments named A and B, comprising active and repressive chromatin, respectively.^91^, ^92^ A‐compartments are decondensed and possess histone marks indicative of transcriptional activation, while B‐compartments possess repressive histone marks.^91^, ^92^ Large regions of heterochromatin (B‐compartment) that reside at the NE and associate with the nuclear lamina are termed lamina associated domains (LADs; Figure 2).^92^, ^93^ It is important to note that LADs are contained within B‐compartments at the NE, but B‐compartments are not exclusively found at the nuclear periphery.^94^

The DNA adenine methyltransferase identification (DamID) technique was used to map protein‐chromatin interactions by expression of DNA adenine methyltransferase fused to a chromatin‐binding protein.^95^, ^96^ Early DamID experiments using emerin or lamin B1 fused to Dam showed both emerin and lamin B1 interact with repressed chromatin regions at the INM.^97^ Using Dam‐lamin B1, LADs were mapped across the human and mouse genomes using various cell types.^93^, ^98^ Dam‐emerin was used in human fibroblasts,^93^ mouse embryonic stem (ES) cells,^99^ and *C. elegans* ^100^ to identify LADs and compare emerin LADs to lamin LADs. Dam‐lamin B1 and Dam‐emerin maps had extensive overlap.^93^ In combination with DamID, a fluorescently labeled m6A‐tracer probe can be used to visualize LAD localization.^101^ The m6A‐tracer probe is based on a restriction enzyme (Dpn1) that has high specificity for G^m6^ATC sequences^102^ but lacks endonuclease activity.^101^ One recent study used the DamID and m6A‐tracer system to track LAD organization throughout mitosis, and showed nuclear lamina proteins are important for post‐mitotic enrichment of LADs at the NE.^103^ Emerin may also be important for post‐mitotic LAD enrichment at the NE as emerin is required for proper nuclear assembly in HeLa cells.^104^ In another recent study using migrating human RPE‐1 cells, Dam‐emerin and m6A‐tracer were employed to show the polarity of emerin‐LAD localization coincides with the polar localization of emerin and repressive histone modifications.^2^

LADs are large genomic regions (0.1–10 megabases) enriched for repressive marks (H3K9me2/3) indicative of a transcriptionally repressive environment.^93^ LAD borders are enriched for the facultative heterochromatin mark H3K27me3,^92^, ^93^ with H3K9me2/3 and H3K27me3 overlapping up to ~200 kb.^92^, ^93^ Peripheral localization of LADs was dependent on H3K9me2/3 and H3K27me3^105^, ^106^ and depletion of H3K9me2 led to LAD loss at the nuclear periphery.^101^ ChIP experiments with H3K9me2 antibodies revealed similar domain profiles to Dam‐lamin B1 experiments,^101^ confirming lamin B1 and H3K9me2 interaction and enrichment in LADs at the NE. Most bulk LADs, named constitutive LADs (cLADs), are gene poor and are highly conserved across cell types and species, with 91% LAD conservation across mouse cell types.^98^, ^107^

Generally, LADs are gene‐poor, but if genes are present in LADs, they are often enriched for developmental and cell identity genes.^93^, ^107^ Developmental gene loci within LADs, which are turned on or off at different stages of development or stem cell differentiation, are termed facultative LADs (fLADs). Lamin B1 DamID of murine ES cells,^108^ neural precursor cells, astrocytes, and embryonic fibroblasts^107^ revealed fLADs are cell‐type specific and their maintenance at the NE controls transcription of lineage genes.^98^ More recently, a study performed lamin B1 ChIP‐seq in 12 cell types derived from human pluripotent SCs and confirmed that fLADs were cell‐type specific.^109^

**FIGURE 3:**
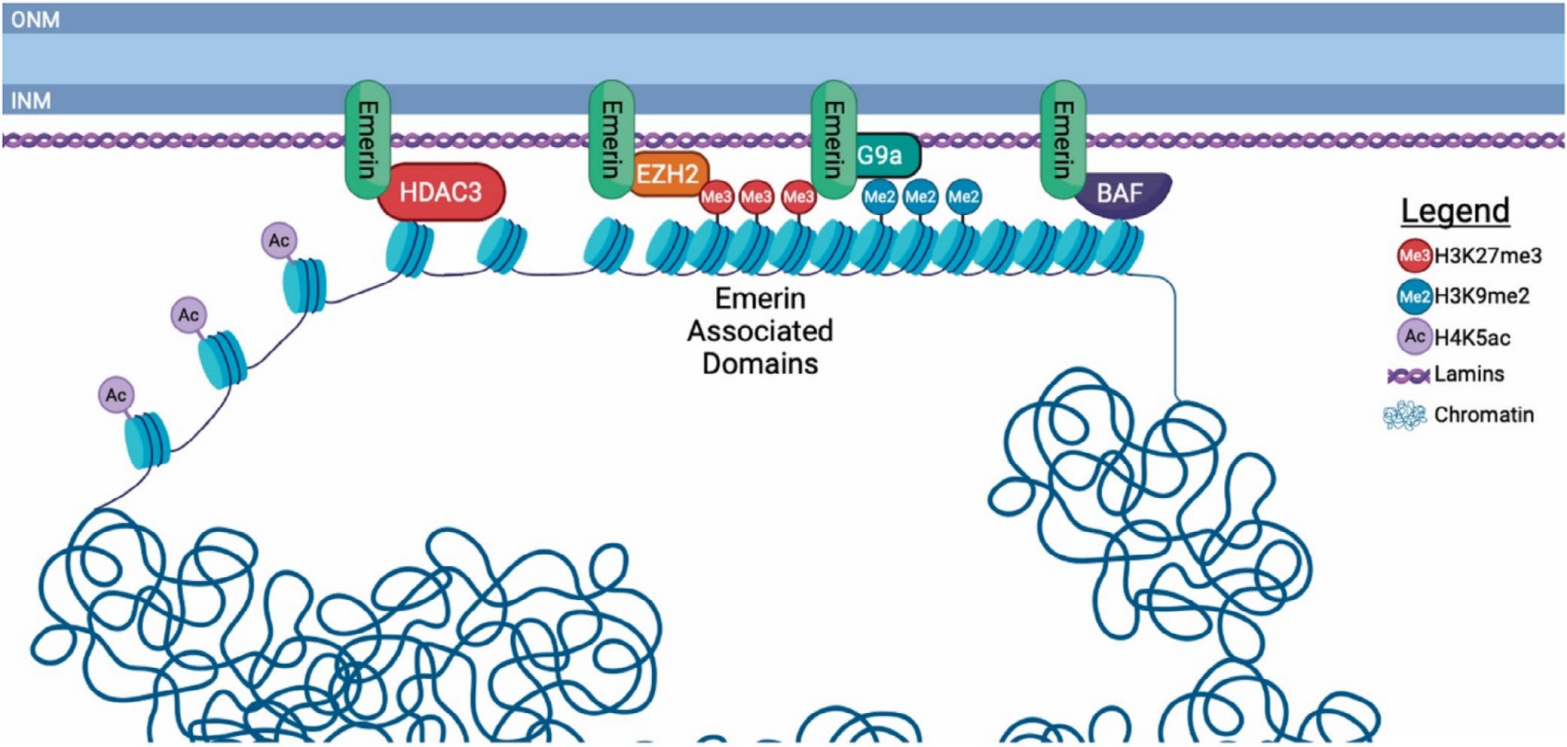
Emerin interacts with histone modification machinery. Emerin (green) binds to HDAC3 (red) and activates its catalytic activity to remove acetylation on H4K5 to repress chromatin. Emerin interacts with the PRC2 complex (orange) and G9a (turquoise) which deposits methylation marks on H3K27 and H3K9, respectively. These methylation marks result in further compaction of the chromatin. Emerin and BAF (purple) may also help to organize chromatin at the nuclear envelope. Figure created with BioRender.com.

Genomic organization at the NE occurs through recruitment and tethering of heterochromatin at the nuclear periphery. Dynamic reorganization of chromatin at the nuclear periphery is a feature of differentiation into specific cell types, including skeletal muscle.^110^ Surprisingly, lamins were reported to be dispensable for LAD formation at the NE,^111^, ^112^ suggesting other INM proteins, including emerin, likely play a role in LAD localization and chromatin tethering at the nuclear periphery. Emerin has been implicated in the recruitment or stabilization of repressive chromatin via association with histone modification machinery. Specifically, emerin interacts with HDAC3 and histone methyltransferases (EZH2 and G9a) that deposit repressive histone modifications (Figure 3).^10^, ^33^, ^113^, ^114^ Emerin binds directly to HDAC3 and activates its catalytic activity.^10^ The emerin‐HDAC3 interaction is important for sub‐nuclear localization of myogenic differentiation loci *MyoD1*, *Myf5*, and *Pax7* to regulate their transcription.^33^ Knockdown of HDAC3 also disrupted LAD localization.^115^ Emerin‐null myogenic progenitors fail to temporally reorganize chromatin architecture when induced to differentiate, which is rescued by activating HDAC3 activity.^33^ Targeting H4K5 acetylation via inhibition of H4K5‐specific histone acetyltransferases also led to a partial rescue of differentiation.^116^ These data support the emerin‐HDAC3 interaction as playing important roles in genomic remodeling during myogenesis.

In addition to increased H4K5ac in emerin‐deficient cells, there are reductions in the transcriptionally repressive marks H3K9me3 and H3K27me3.^10^ Emerin also binds EZH2, either directly or through Msx1, to inhibit the expression of myogenic transcription factors.^113^, ^117^ EZH2 is important for chromatin organization at the NE, as EZH2 downregulation led to the loss of peripheral localization of LADs.^105^ EZH2 is the catalytic component of PRC2 that methylates H3K27^118^ and promotes myogenic differentiation.^119^, ^120^ Pharmacological inhibition of EZH2 also inhibited the differentiation of myogenic progenitors.^114^

How the interactions between emerin, HDAC3, and HMTs are dynamically regulated to modulate differentiation remain to be defined, but growing evidence supports their dynamic interactions during differentiation. For example, emerin mutants that fail to bind HDAC3 (S54F, Q133H) bound significantly more EZH2 and G9a than wild‐type emerin,^114^ suggesting HDAC3 and HMTs compete with one another for binding to emerin. These mutants also had greater peripheral localization of both H3K9me2 and H3K27me3.^114^ However, it is unclear how emerin binding to HMTs regulates dynamic chromatin organization at the NE. In one possible scenario, HDAC3 and HMTs compete for binding to emerin, and thus stronger interactions of HMTs are seen with emerin mutants that fail to bind HDAC3. In this model, unacetylated H4K5 is normally required to define fLADs, which along with other undefined modifications, keeps them primed for rapid activation or repression dependent upon differentiation signals. Alternatively, H4K5 deacetylation and HDAC3‐emerin‐mediated recruitment of chromatin to the NE may serve to initiate fLAD formation, in which case HMTs would then be recruited to emerin‐bound chromatin to be more stably repressed by HMTs. Here the EDMD1‐causing emerin mutants would disrupt transcriptional reprogramming by disrupting the organization of fLADs (*Pax3/7, Myf5*, and *MyoD1*) through disrupting H4K5, H3K9me2/3, and H3K27me3 homeostasis due to a lack of HDAC3 binding. Alternatively, it is possible that increased emerin‐HMT interactions in the presence of HDAC3‐binding emerin mutants act to compensate for aberrantly activated cell‐cycle genes, lineage‐specific genes, and/or terminal differentiation genes. Another model is that HDAC3 serves as a chromatin tether, and its catalytic activity is not required for fLAD formation and/or localization, as was shown in cardiomyocytes.^121^ Similarly, HMTs may serve as chromatin tethers at the INM and the effect of HDAC3‐binding emerin mutants is due simply to them binding more HMTs at the NE, irrespective of HDAC3.

More recently, other techniques have been developed to interrogate protein–protein interactions. The BioID technique relies on a modified biotin ligase fused to a bait protein.^122^ Proteins proximal to the bait in the presence of biotin are biotinylated and isolated for the identification of protein–protein interactions. This approach was used in MEFs with emerin and LBR in one study^53^ and LAP2β in another.^123^ Emerin and LAP2β interact with each other and share several interactors including SUN1, SUN2, and MAN1 but do not share other interactors such as B‐type lamins, which were only identified in LAP2β BioID experiments.^53^, ^123^ Emerin and LBR also share binding partners but do not completely overlap.^53^ The micro‐proteome maps of emerin, LAP2β and LBR, while shared to a degree, also support a model in which INM proteins function and interact with distinct but overlapping complexes. BioID was also combined with the m6A‐tracer system by fusing the biotin ligase to the m6A‐tracer. In this system, Dam‐lamin B1 methylates lamina‐associated chromatin to be recognized by the m6A‐tracer fusion protein, which leads to the biotin‐labeling of proximal LAD proteins.^123^ The authors defined three interaction zones at the nuclear periphery. Zone 1 corresponded to INM/lamina proteins that did not interact with LADs. Zone 2 corresponded to proteins that interacted with both INM/lamina proteins and LADs. Zone 3 corresponded to proteins that were restricted to LADs. Emerin was described as a zone 2 protein, where it functions as a “middle‐man” by interacting with nuclear lamina proteins and chromatin.^123^ Collectively, these data show emerin plays a vital role in genomic organization at the nuclear lamina–LAD interface.

## 5 EMERIN REGULATION OF CELL SIGNALING

Evidence supports the theory that EDMD1 pathology derives from defective differentiation caused by loss of emerin function in genomic organization,^33^, ^114^ transcriptional regulation^8^, ^11^ and cell signaling.^124^, ^125^ Genome‐wide mRNA microarray analysis of skeletal muscle from emerin‐null mice and EDMD patients, and myogenic progenitors derived from emerin‐null mice, revealed disruptions in various signaling pathways critical for myogenic differentiation.^124^, ^125^ These included upregulation of Retinoblastoma protein (Rb)‐MyoD pathway components (CREBBP, EP300, and CRL‐1),^124^, ^125^ which are important regulatory components of the myogenic differentiation program.^126^, ^127^ Perturbations in the expression of Rb/MyoD pathway components were also observed in muscle biopsies of EDMD1 (*EMD*) and EDMD2 (*LMNA*) patients.^124^ RNA sequencing on differentiating wild type and emerin‐null myogenic progenitors showed that the expression of more than 3000 genes was altered in differentiating emerin‐null myogenic progenitors.^11^, ^27^ Signaling pathways altered in differentiating emerin‐null progenitors overlapped with those identified in emerin‐null mice and EDMD patient skeletal muscle,^124^, ^125^ including ERK, Rb, Notch, Wnt, and TGF‐β pathways. They also identified new putative pathways, including HIPPO signaling, VEGF signaling, G2/M signaling, and others.^11^, ^27^ Thus, disruptions in these transcriptional and cell signaling pathways are likely major contributors to the impaired myogenic differentiation observed in emerin‐null progenitors.^11^, ^27^, ^114^

**FIGURE 4:**
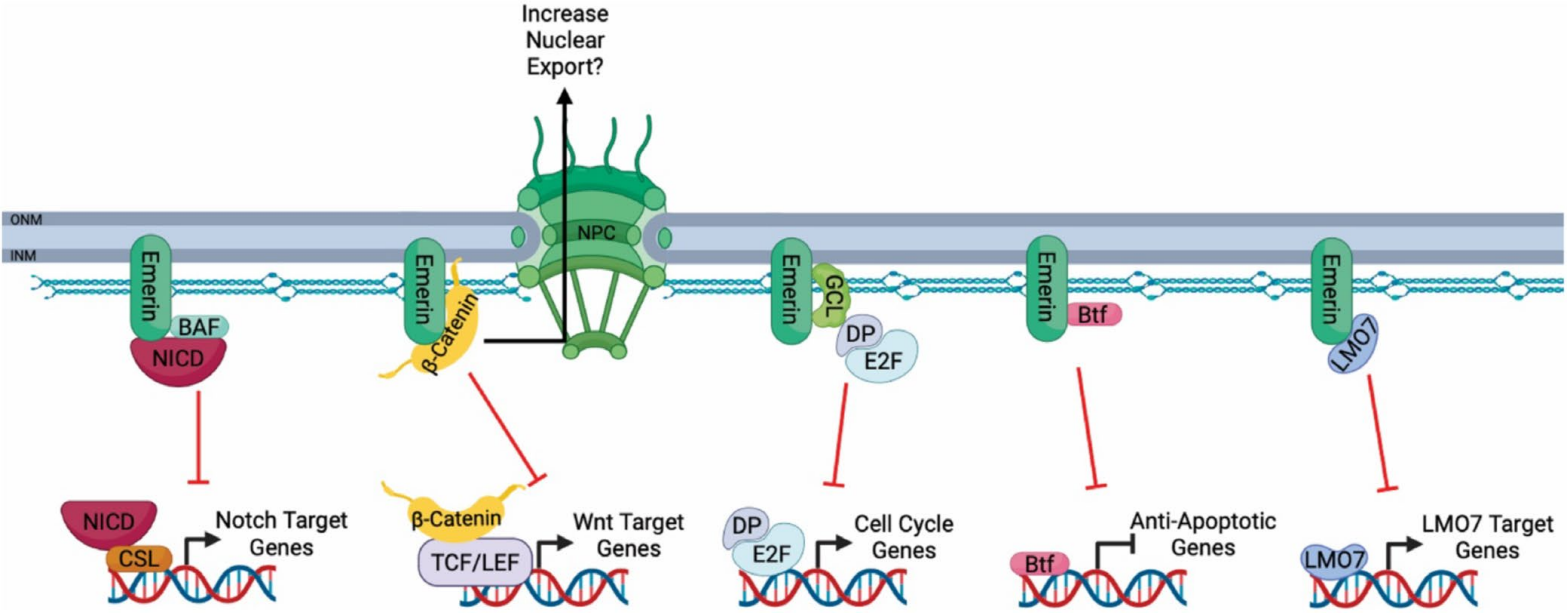
Emerin binds transcription regulators at the nuclear envelope. Emerin modulates transcription of Notch target genes by restricting NICD (maroon) to the nuclear envelope with BAF (aqua). Emerin prevents nuclear accumulation of β‐catenin (yellow) to prevent transcription of Wnt target genes. Emerin binds to GCL (green) and indirectly sequesters DP/E2F at the NE to prevent transcription of E2F target genes. Emerin binds Btf (pink) to prevent transcriptional repression of anti‐apoptotic genes. Emerin binds to Lmo7 (blue) to prevent transcription of Lmo7 target genes, including *MyoD1* and *Pax3*. Figure created with BioRender.com.

Transcriptional profiling of emerin‐null myogenic progenitors showed increased Notch signaling,^12^ which likely contributes to the EDMD1 phenotype by attenuating the differentiation program. The Notch signaling pathway is evolutionarily conserved and is implicated as an important regulator of myogenic differentiation.^128^ Canonical Notch signaling occurs through Notch ligand binding to Notch transmembrane receptors at the plasma membrane, resulting in cleavage of the receptor and release of Notch intracellular domain (NICD) to activate Notch target gene expression.^129^ Emerin and BAF bind NICD at the nuclear periphery and inhibit the transcriptional activation of Notch target genes (Figure 4).^7^ By inhibiting Notch signaling, emerin thereby promotes myogenic differentiation.

A switch from Notch to Wnt signaling is required for differentiation.^130^ Wnt signaling is important for myogenic differentiation and for repairing damaged skeletal muscle.^130^, ^131^ Canonical Wnt signaling regulates ES cell differentiation through the activation of β‐catenin transcriptional activity.^132^ Emerin attenuates β‐catenin activity in murine ES cells.^9^ Emerin inhibited β‐catenin activity in HEK293 cells by restricting its accumulation in the nucleus, as loss of emerin resulted in greater nuclear accumulation of β‐catenin.^8^ It will be important to define whether emerin acts by increasing β‐catenin nuclear export or by inhibiting its nuclear import.

Insulin‐like growth factor 1 (IGF‐1) signaling has roles in autophagy, proliferation, migration, and cellular identity.^133^ IGF‐1 signaling also has roles in myogenesis.^134^, ^135^ We found IGF‐1 signaling is enhanced in proliferating emerin‐null myogenic progenitors.^11^, ^12^ This increase likely promotes myogenesis. However, emerin‐null myogenic progenitors also exhibited attenuated TGF‐β signaling,^12^ which is predicted to promote differentiation, as transforming growth factor‐beta (TGF‐β) signaling was shown to be an inhibitor of myogenic differentiation.^136^, ^137^, ^138^ We propose the decreased TGF‐β signaling that simultaneously occurs with IGF‐1 signaling in emerin deficiency^12^ depletes the stem cell niche. How IGF‐1 and TGF‐β pathways functionally interact to contribute to impaired myogenic differentiation in the absence of emerin remains to be defined.

Emerin is implicated in regulating the G1/S cell cycle checkpoint by regulating Rb signaling.^27^, ^125^ Rb is a tumor suppressor that controls cell‐cycle progression in a phosphorylation‐dependent manner.^139^, ^140^ When Rb is hyperphosphorylated by cyclin‐dependent kinases (CDKs), Rb dissociates from E2F/DP, and E2F/DP‐regulated genes (e.g., Cyclin E) are transcribed, allowing progression from G1‐ to S‐phase.^141^

Rb has direct roles in skeletal muscle differentiation by interacting with myogenic transcription factors to regulate the myogenic differentiation program.^142^, ^143^ One such interaction is with MyoD. The role of MyoD signaling in lineage commitment and cellular identity has been well established.^144^, ^145^, ^146^ MyoD interacts with HDAC1, and Rb can recruit HDAC1 from MyoD.^147^ This allows for MyoD acetylation and its subsequent activation to promote myogenic differentiation.^147^ Rb and MyoD pathway components are upregulated by loss of emerin in EDMD patients.^124^ Myoblasts from emerin‐null mice showed similar disruptions in Rb/E2F and MyoD signaling, which caused a delay in cell cycle withdrawal and inhibition of myotube fusion.^125^ Additionally, MyoD was shown to activate Notch signaling by activating the expression of Notch ligand Delta‐like canonical notch ligand 1 (Dll1),^148^ illustrating the interplay between these signaling pathways during myogenic differentiation.

Emerin also directly binds to several other transcriptional partners.^149^ Emerin binds directly to germ‐cell less (GCL).^64^ GCL binds the DP subunit of the E2F transcription factor and inhibits transcription of E2F target genes.^150^ Emerin binding to GCL is predicted to release E2F‐DP from GCL to activate cell cycle genes that drive progression through the G1/S checkpoint. The same emerin domains, named regulatory binding domains‐1 and ‐2 (RBD‐1 and RBD‐2) that bind GCL also bind Bcl2‐associated transcription factor (Btf).^63^ Emerin binds to LIM‐domain only 7 (Lmo7) and inhibits Lmo7 binding to *Pax3* and *MyoD1* promoters^65^, ^151^ resulting in impaired differentiation. Lmo7 is also a negative regulator of TGF‐β signaling,^152^ so how the interaction between emerin and Lmo7 affects TGF‐β signaling needs to be defined. Interestingly, Lmo7‐null mice displayed a phenotype consistent with EDMD1, with dysfunctions in skeletal muscle and cardiac function.^153^ Another Lmo7‐null mouse model showed no defects in skeletal muscle function or cardiac function, but the reasons are unclear.^154^

## 6 EMERIN REGULATION OF NUCLEAR STRUCTURE AND MECHANOTRANSDUCTION

The nuclear lamins are integral in maintaining nuclear structure and organization of INM proteins.^155^ It is predicted that emerin reinforces nuclear structure and rigidity in cooperation with lamins, other INM proteins, and nuclear F‐actin.^62^, ^155^ In MEFs, the lack of emerin or lamin A/C resulted in nuclear morphology abnormalities and mechanical deficiencies.^5^, ^156^ Another study using emerin‐null myogenic progenitors and a cancer cell line with low emerin expression (MDA‐231) showed reductions in nuclear area.^1^ shRNA‐mediated knockdown of emerin in MCF7 cells also showed reductions in nuclear structure.^157^

The interaction of emerin with nuclear structural partners supports a role of emerin in nuclear structure and stiffness. In addition to emerin binding to nuclear lamins, emerin also binds nuclear F‐actin to reinforce nuclear structure.^62^ Nuclear actin regulates nuclear architecture in *Xenopus* oocytes through its association with nuclear pore‐linked filaments and INM proteins, such as emerin.^158^ Emerin binds to the pointed end of short nuclear actin filaments to reinforce the nucleoskeleton.^62^, ^159^ The structural role of emerin binding to nuclear actin is posited to help maintain cellular homeostasis.^160^ Emerin also binds other structural proteins, including spectrin isoform ⍺‐II,^161^ nuclear protein 4.1R^162^ and nuclear myosin I.^161^ Work in a colorectal adenocarcinoma line (DLD‐1) showed emerin knockdown by siRNA disrupted nuclear myosin I localization,^13^ illustrating a role for emerin in nucleoskeletal organization.

Physical stimuli from the cell surface are sensed by adhesion proteins in the plasma membrane and are relayed to the nucleus to elicit cellular responses in a process called mechanotransduction.^163^ Force transmission from the plasma membrane to the nucleus occurs through the LINC complex^164^ to modulate various cellular processes, including chromatin architecture^165^, ^166^ and gene expression.^167^, ^168^ The LINC complex consists of KASH (Klarsicht/ANC‐1/SYNE homology)‐domain proteins named nesprins (nuclear envelope spectrin repeat proteins) and SUN‐domain proteins.^169^ Nesprin 1 is a large protein (1000 kDa) composed primarily of spectrin repeat domains that adopts a long, alpha‐helical structure.^170^ Nesprin 1 binds to cytoskeletal actin via its N‐terminal calponin homology domain.^170^ It then binds to the SUN‐domain proteins in the periplasmic space between the INM and ONM via its KASH domain on its C‐terminus.^171^, ^172^ The SUN‐domain proteins are integral INM proteins that form a homotrimeric complex.^173^ SUN1 was initially identified in proteomic screenings as a homolog to *C. elegans* UNC‐84^174^ and also has homology to *S. pombe* Sad1.^173^ The N‐terminus of SUN1 extends into the nucleoplasm where it can bind to lamins^175^ and directly to emerin.^53^, ^54^

In skeletal muscle, the LINC complex is important in myonuclei positioning^176^ and chromatin repression.^167^ Perturbation of the LINC complex resulted in nuclear deformities,^177^ chromatin relaxation,^177^ myofiber nuclear positioning disruptions,^178^ and fibrosis.^179^ In *C. elegans*, the nesprin homolog, ANC‐1, was shown to mediate the positioning of muscle nuclei in coordination with actin and the SUN1 homolog, UNC‐84.^180^ Similar observations were seen in both human and mouse myoblasts.^181^ Nuclear movement and positioning are also controlled by transmembrane actin‐associated nuclear (TAN) lines, which are a structural feature of the NE composed of LINC components nesprin‐2G and SUN2.^182^ In MEFs, knockdown of nesprin‐2G or SUN2 disrupted TAN line structure and association with actin cables, leading to reductions in cell migration and disruptions in nuclear positioning.^183^ Lamin A/C expression is also critical for TAN line formation and function, as migration and centrosome positioning and orientation were impaired in *LMNA* ^*−/−*^ murine embryonic fibroblasts.^184^ Emerin loss was shown to depolarize actin cables and disrupt TAN line movement in fibroblasts.^185^ Thus, proper emerin and LINC function play an important role in TAN‐line mediated nuclear positioning. The importance of LINC mechanotransduction for skeletal muscle function is also illustrated by the fact that mutations in LINC components are linked to EDMD.^186^

Emerin is implicated in mechanotransduction^4^, ^187^ through its interaction with the LINC complex.^188^ Emerin directly interacts with SUN1/2 and nesprins.^28^, ^54^, ^55^ Interestingly, emerin knockdown by RNAi disrupted nesprin‐1 and SUN2 localization, while nesprin‐2 and SUN1 localization remained unaffected.^2^ It is likely that loss of emerin compromises nuclear integrity and affects force transmission conferred by the LINC complex. This was shown in emerin‐null MEFs^5^ and emerin‐null fibroblasts,^4^ where there was an increase in nuclear dysmorphism and reduced mechanotransduction signaling in the absence of emerin. We predict that disrupting emerin and LINC binding will inhibit robust cellular responses to mechanical stimuli by failing to reorganize the genome, resulting in aberrant transcriptional responses that have profound effects on muscle regeneration and function.

## 7 CONCLUSIONS AND FUTURE DIRECTIONS

Emerin loss‐of‐function or nonsense mutations disrupt the tightly regulated myogenic differentiation program, leading to inefficient muscle regeneration. Emerin serves as a critical regulator of INM organization, gene expression, and LAD organization. LADs are cell‐type specific and undergo dynamic reorganization during differentiation, a process that depends on emerin in muscle progenitor cells. Through its interactions with other INM proteins, emerin coordinates genome organization at the NE, where it facilitates the temporal activation or repression of stem cell and myogenic gene loci within fLADs. However, the exact mechanism by which emerin regulates chromatin remains unclear. It is still unknown whether emerin achieves this through recruitment of chromatin modifiers, such as HDAC3 or HMTs, or whether its primary function is to tether repressive chromatin at the NE.

In addition to its role in chromatin organization, emerin binding to nuclear structural proteins likely plays a significant role in myogenic differentiation. Nuclear architecture and genomic organization are intimately connected and influenced by mechanical forces. Emerin interacts with components of the LINC complex, a central player in cellular mechanotransduction, allowing it to directly influence the nucleoskeleton and mechanosensitive nuclear organization. This connection highlights emerin's ability to integrate mechanical cues with genomic regulation, a process that is essential for muscle development and regeneration.

Patient‐derived EDMD1 mutations that are expressed at near‐wild type levels and localize correctly to the INM offer a powerful tool to dissect emerin function. These ‘special’ mutations provide a unique opportunity to identify which specific functions or binding partners of emerin are critical for myogenic differentiation. Moving forward, the use of EDMD1 patient‐derived induced pluripotent stem cells represents an important advance, as these cells offer a physiologically relevant model for studying human myogenic differentiation and disease mechanisms. This approach will help overcome some of the limitations of murine models.

While existing research highlights transcriptional reprogramming deficiencies because of emerin loss, the next step will be to investigate bulk chromatin interactions at the nuclear periphery throughout differentiation. A comprehensive analysis of large‐scale chromatin organization in differentiating wild type and EDMD1 skeletal muscle cells combined with transcriptional profiling will be essential to fully elucidate emerin's role in myogenic differentiation. This integrative approach will provide critical insights into how emerin regulates nuclear architecture, chromatin dynamics, and gene expression programs during muscle development and regeneration.

## AUTHOR CONTRIBUTIONS

Nicholas Marano and James M. Holaska conceived review article topics; Nicholas Marano and James M. Holaska designed figures; Nicholas Marano and James M. Holaska wrote, edited, and revised the manuscript.

## FUNDING INFORMATION

NIH R15AR069935.

## DISCLOSURES

The authors declare no conflicts of interest.

## Data Availability

No new data. Data sharing is not applicable to this article as no datasets were generated or analyzed during the current study.
